# Management of sterile abdominal pseudocysts in the context of ventriculoperitoneal shunts: a systematic review

**DOI:** 10.1007/s00381-025-06823-3

**Published:** 2025-04-15

**Authors:** A. Shehaj, T. Harbaugh, H. Wilding, M. Mareboina, M. Newland, E. Rizk

**Affiliations:** Department of Neurosurgery, Penn State College of Medicine, 700 HMC Cres Rd, Hershey, PA 17033 USA

**Keywords:** Abdominal pseudocyst, Ventriculoperitoneal shunt, Hydrocephalus, Pediatric hydrocephalus

## Abstract

This systematic review investigates the management of sterile abdominal pseudocysts (APCs), a complication associated with ventriculoperitoneal shunts (VPS). Management options for sterile APCs include repositioning and externalization, but there remains no consensus on which management technique is superior in terms of outcomes in recurrence or overall complication rates. Therefore, a comparison of outcomes between shunt externalization and repositioning was conducted. A systematic review was done through PRISMA guidelines, and a search of multiple databases, including Medline and Embase, was conducted from the date of inception until 2023. The search results demonstrated 382 articles. Of the 382 articles, 252 were unique, and 43 articles were included in the analysis after full-text analysis. The results of our analysis indicate there is no significant difference in pseudocyst recurrence and overall complications between externalization and repositioning of the distal shunt catheter. The rate of pseudocyst recurrence for studies with a follow-up of 6 or more months was 25% and 24.1% for repositioning and externalization, respectively (*p* = 0.99). The overall complication rate for studies with a follow-up of 6 or more months was 44.4% and 34.5% for repositioning and externalization, respectively (*p* = 0.3861). Although our analysis did not demonstrate a significant difference between the two approaches, further work that includes prospective studies, longer follow-up periods, and larger sample sizes is needed to establish this.

## Introduction

Cerebrospinal fluid (CSF) shunt placement is the primary form of management for pediatric hydrocephalus [1, 2]. The ventriculoperitoneal shunt (VPS) is the predominant shunting system frequently utilized to treat hydrocephalus and function by shunting excess CSF from the cerebral ventricles and diverting it to the peritoneal cavity, where it is absorbed, relieving elevated intracranial pressure due to hydrocephalus [3, 4]. Despite their therapeutic utility in pediatric and adult hydrocephalus, these shunts can lead to complications, such as abdominal pseudocysts, and frequently require revisions [2]. It has been demonstrated that over 80% of patients with shunts need at least one revision throughout their lifetime [5]. Due to this, it is essential for clinicians to understand how to appropriately manage these patients and the complications of VPS to minimize the need for further revisions.

**Fig. 1:**
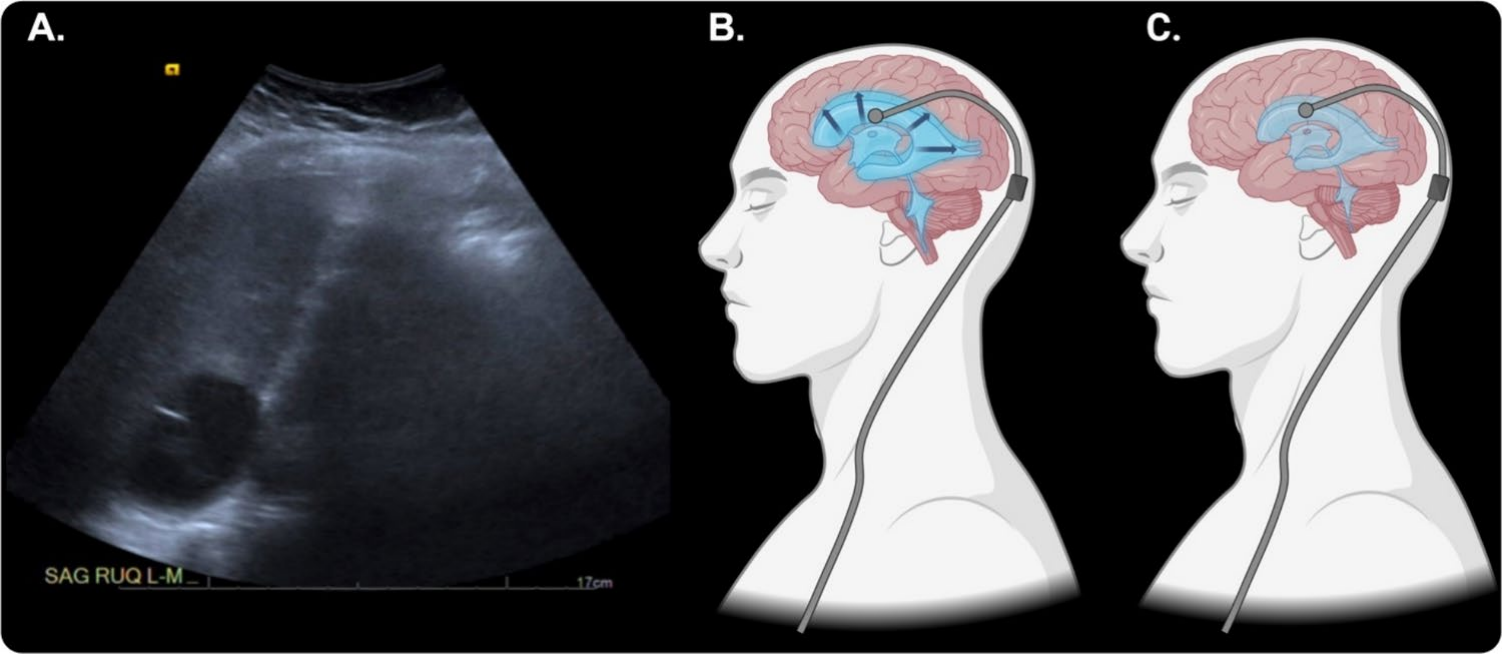
Illustration of increased ventricular CSF due to shunt failure from an APC with subsequent resolution after repositioning or externalizing the distal catheter. **A** Depiction of an abdominal pseudocyst in the right upper quadrant. **B** Distal shunt failure caused by the APC leading to increased ventricular CSF. **C** Shunt function is restored after externalization or repositioning of the distal catheter (created through BioRender)

Abdominal pseudocysts (APCs) are a common abdominal complications related to VPS, which have been reported with an incidence rate of 0.25 to 10% [1, 6, 7]. They typically present with abdominal pain, distention, and fever. Current recommendations for evaluating APCs involve obtaining fluid samples to confirm sterility. If an abdominal pseudocyst is non-sterile, it should be appropriately managed by shunt externalization, antibiotics, and eventual re-internalization. However, recommended management options for sterile APCs vary and include either externalizing the distal shunt with later internalization or repositioning the distal catheter in the peritoneum [8]. Since APCs can lead to shunt failure and eventual increase in CSF, it is essential to understand which approach is most appropriate to amend the failure and mitigate any future complications, as demonstrated by the illustration in Fig. 1. There remains no consensus on which management technique is superior regarding recurrence, complication rates, or further revisions needed. Furthermore, studies comparing the management of sterile APCs through externalizing VPS with later internalization versus repositioning the distal catheter are scarce. This study aimed to conduct a systematic and comprehensive review of the existing literature regarding these two management techniques. This study serves the goal of helping clinicians determine which management technique they should consider when caring for patients with abdominal pseudocysts as a complication of VPS. Our secondary goal included assessing trends in the publication of articles related to APC management.

## Methods

### Search strategy

A systematic review was conducted in accordance with the Preferred Reporting Items for Systematic Reviews and Meta-Analyses (PRISMA) Statement for Reporting Systematic Reviews [9]. A comprehensive computerized literature search was conducted through Medline/Pubmed and Embase/Scopus without date limits until the end of 2023. These databases were queried for potential studies using the terms (“sterile abdominal pseudocyst” OR “pseudocyst”) AND (“ventriculoperitoneal shunt”) AND (“Treatment” OR “Management” OR “Complications” OR “Outcomes”). The results from our search are summarized in Table 1. A prospective registration was not performed for this study.

**Table 1:** Database search strategy and results

Database	Search terms	No. of articles
Medline/Pubmed	(“sterile abdominal pseudocyst” OR “pseudocyst”) AND (“ventriculoperitoneal shunt”) AND (“Treatment” OR “Management” OR “Complications” OR “Outcomes”)	136
Embase/Scopus	(“sterile abdominal pseudocyst” OR “pseudocyst”) AND (“ventriculoperitoneal shunt”) AND (“Treatment” OR “Management” OR “Complications” OR “Outcomes”)	246

### Inclusion and exclusion criteria

Articles for potential selection were screened through various inclusion and exclusion criteria. Inclusion criteria included studies published in peer-reviewed journals discussing the management of sterile abdominal pseudocysts related to ventriculoperitoneal shunts. Studies with the following criteria were excluded: (1) non-English; (2) basic science studies; (3) animal studies; (4) review papers; (5) not a full-text article (letters or editorials); (6) shunt conversion (e.g., conversion to ventriculoatrial shunt); (7) APCs were non-sterile (8) no outcome measures were reported or no mention of post-operative condition. Two authors from the A.S., T.H., H.W., and M.M. list independently reviewed each study potentially included in the systematic review. Disagreements on the inclusion of a study were resolved by a third author who did not initially review the study in question. Manual searches of review bibliographies and reference lists of primary studies that were not captured by the electronic searches were performed.

### Data extraction

Data extraction was performed by a team of four authors (A.S., T.H., H.W., and M.M.). Data extracted from selected articles encompassed publication details (authors, publication year, and study design); demographic information (age and sex); follow-up duration; rate of pseudocyst reoccurrence; rate of needing further revisions; and any antibiotic usage.

### Statistical analysis

Descriptive statistics, including frequencies and percentages, were used to report demographic and clinical outcomes. Frequencies of pseudocyst reoccurrence, subsequent shunt conversions, and antibiotic usage were gathered. An analysis was conducted for the overall data, and a separate analysis was conducted only for studies with a follow-up of 6 or more months. Lastly, trends regarding the occurrence and management of abdominal pseudocysts were investigated by plotting the number of published studies and patients treated from the date of inception until the end of 2023.

## Results

### Study selection

**Fig. 2:**
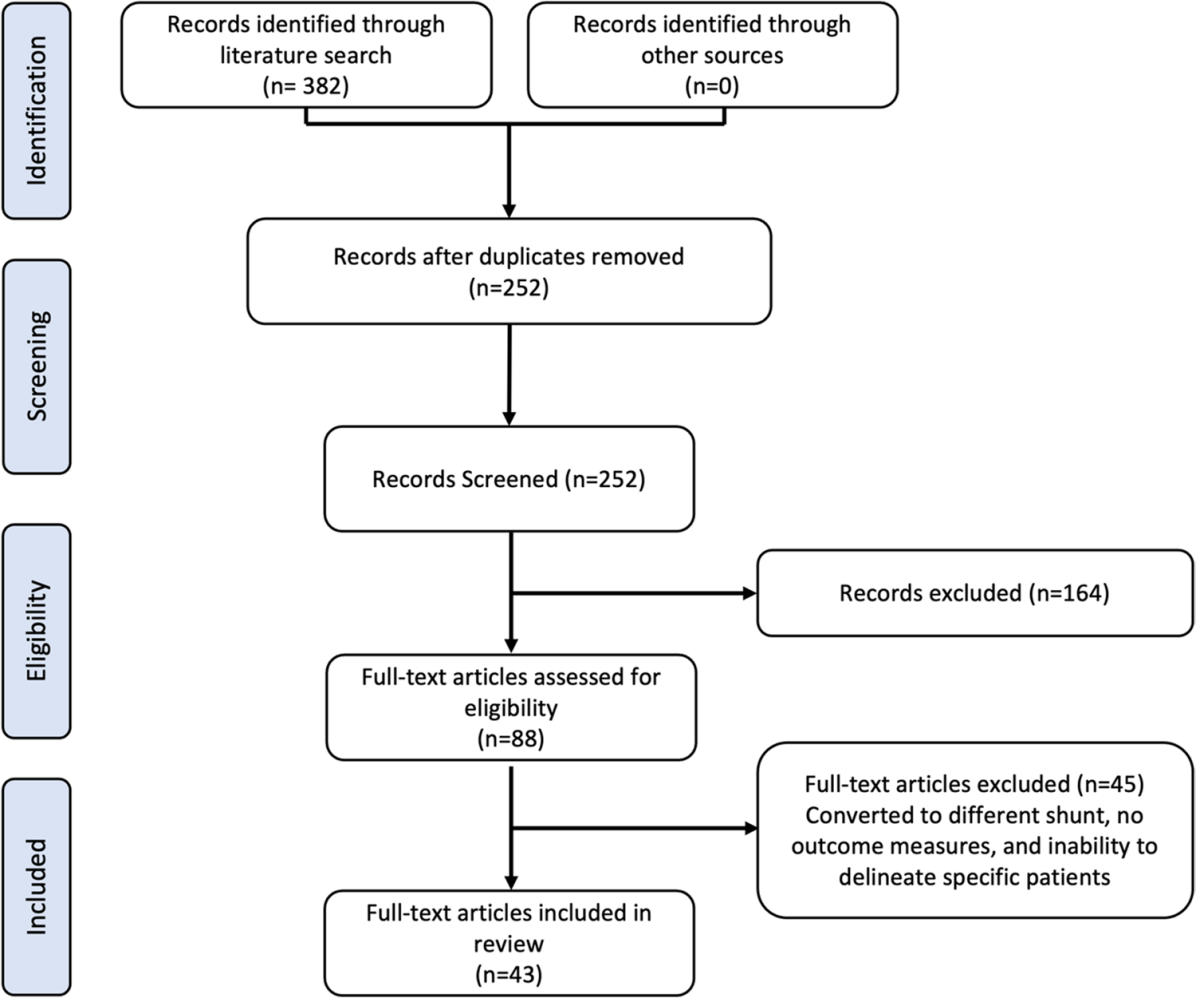
PRISMA flow diagram for study selection. Study selection outline demonstrating 382 search results with 252 studies after duplicate removal. Through abstract screening, 164 articles were excluded, and 88 full text articles were analyzed. After full text assessment, 45 articles were excluded due to conversion to a different shunt, no outcome measures, and inability to delineate patients. A total of 43 articles were included in the final review

A systematic literature review yielded 382 articles, of which 252 were unique. In total, 252 unique publications were deemed potentially suitable for inclusion and were retrieved and analyzed by two reviewers for final eligibility determination. After abstract screening and full-text review, 164 articles were excluded from the analysis for reasons outlined previously. Forty-three articles met the inclusion criteria, yielding 128 patients for analysis [1, 10–52]. Seventy-nine percent of the articles included were case reports or series with most of the studies having less than ten patients for each respective management approach. The study selection and results are outlined in Fig. 2

### Patient characteristics

A total of 128 distinct patients were identified in the literature to have undergone repositioning or externalization with subsequent reinternalization after an occurrence of sterile abdominal pseudocyst from inception until 2023. Patient ages at the time of surgery ranged from neonates to 59 years old at the time of surgery. Patient sex and demographic clinical information are summarized in Table 2.

**Table 2:** Demographics of patients

Characteristic	No. of patients*	Average age in years (+/− SD)
Sex		
Repositioning	63	19.54 (SD 21.18)
Male	21	
Female	26	
Externalization	64	27.5 (SD 18.37)
Male	14	
Female	13	
Total reported	74	22.0 (SD 20.42)

*SD* standard deviation. *Not all studies reported the breakdown of the patient population by sex

Sixty-two patients met the criteria for the repositioning group. Of the 62 patients, 14 were determined to have a pseudocyst reoccurrence (22.6%). The overall rate of complications for the repositioning group was 37.1% (23/62). The pseudocyst recurrence rate and overall complications for studies with a follow-up of 6 or more months were 25% and 44.4%, respectively. All findings were determined to be statistically insignificant, as determined by Fisher’s exact test. The selected studies by author and year, study type, number of patients, frequency of APC reoccurrence, and overall complications are highlighted in Table 3. Overall complications include APC recurrence, further shunt revisions, conversion to an alternate shunt type, or shunt infection. Further shunt revisions and infections were also noted for some patients.

**Table 3:** Selected studies for ventriculoperitoneal distal catheter repositioning

Author, year	Study type	Follow-up (months)	No. of patients	Reoccurrence	Overall complications
Dzongowski et al. 2017 [10]	Retro	*	4	2	3
Hamid et al. 2017 [11]	Retro	12	3	0	0
Burhan et al. 2018 [12]	Retro	12	1	0	0
Fatani et al. 2020 [13]	Case Series	8	1	1	1
**Mobley et al. 2005 [14]	Retro	6	7	4	4
**Gmeiner et al. 2018 [1]	Retro	240	4	1	4
Masoudi et al. 2017 [15]	Case report	6	1	1	1
Kumar et al. 2022 [16]	Case Series	12	1	0	1
Tamura et al. 2013 [17]	Case Report	4	1	0	0
Dutta et al. 2018 [18]	Case series	6	1	0	0
Aparici-Robles and Molina‐Fabrega 2008 [19]	Case Series	6	5	1	1
Parelkar et al. 2014 [20]	Case report	6	1	0	0
Coley et al. 2004 [21]	Case Series	12	5	0	1
Baumgartner et al. 1990 [22]	Case report	*	1	1	1
Jain et al. 2003 [23]	Case report	8	1	0	1
Kim et al. 1995 [25]	Case report	12	1	0	0
Kim et al. 2016 [24]	Case Report	*	1	0	0
Cunningham et al. 2014 [26]	Case report	*	1	0	0
Canaz et al. 2017 [27]	Case series	9	2	0	0
**de Oliveira et al. 2006 [28]	Case series	108	1	1	1
Kumar et al. 2003 [29]	Case series	*	1	0	0
Handa et al. 2005 [30]	Case report	*	1	0	0
Agarwal et al. 2009 [31]	Case report	*	1	0	0
**Briggs et al. 1984 [32]	Case series	*	3	0	0
Palomar et al. 1977 [33]	Case series	*	2	1	2
Fábrega et al. 2006 [34]	Case report	*	1	0	0
Guice 1978 [35]	Case Report	*	1	1	1
Sena et al. 2010 [36]	Case Report	1	1	0	0
Bartolek et al. 2010 [37]	Case Report	*	5	1	1
Shrestha et al. 2023 [38]	Case Report	30	1	0	1
Kaplan et al. 2007 [39]	Case Report	*	1	0	0
Peltier et al. 2011 [52]	Case report	7 days	1	0	0

**Manuscript contains data for both externalization and repositioning. *Not reported

Sixty-four patients met the criteria for the externalization group. Out of the 64 total patients, 14 were determined to have a pseudocyst reoccurrence (21.9%). The overall complication rate for the externalization group was 31.3% (20/64). The pseudocyst recurrence rate and overall complications for studies with a follow-up of 6 or more months were 24.1% and 34.5%, respectively. All findings were determined to be statistically insignificant, as determined by Fisher’s exact test. The selected studies by author and year, study type, number of patients, frequency of APC reoccurrence, and overall complications for the externalization group are highlighted in Table 4.

**Table 4:** Selected studies for ventriculoperitoneal distal catheter externalization

Author, year	Study type	Follow-up (months)	No. of patients	Reoccurrence	Overall complications
Erwood et al. 2019 [40]	Retro	12	11	2	2
Koide et al. 2019 [41]	Case report	*	1	0	0
**Mobley et al. 2005 [14]	Retro	6	16	9	9
**Gmeiner et al. 2018 [1]	Retro	240	4	0	0
Krassoudakis et al. 2002 [42]	Case report	12	1	0	0
Nakano et al. 1994 [43]	Case report	12	1	0	0
Arsanious and Sribnick 2019 [44]	Case report	1.5	1	0	0
**Santos De Oliveira et al. 2006 [28]	Case series	96	4	1	1
Verma et al. 2012 [45]	Case report	0.75	1	0	0
Latchaw and Hahn 1981 [46]	Case report	6	1	0	1
Gebarski et al. 1984 [47]	Case report	*	1	0	0
**Briggs et al. 1984 [32]	Case series	18	1	0	0
Roitberg et al. 1998 [48]	Retro	180	10	1	2
White et al. 1991 [49]	Retro	77	2	0	1
Arnell and Olsen 2004 [7]	Retro	288	7	1	4
Risfandi et al. 2022 [50]	Case Report	*	1	0	0
Sebastian et al. 2018 [51]	Case report	3	1	0	0

**Manuscript contains data for both externalization and repositioning. *Not reported

**Fig. 3:**
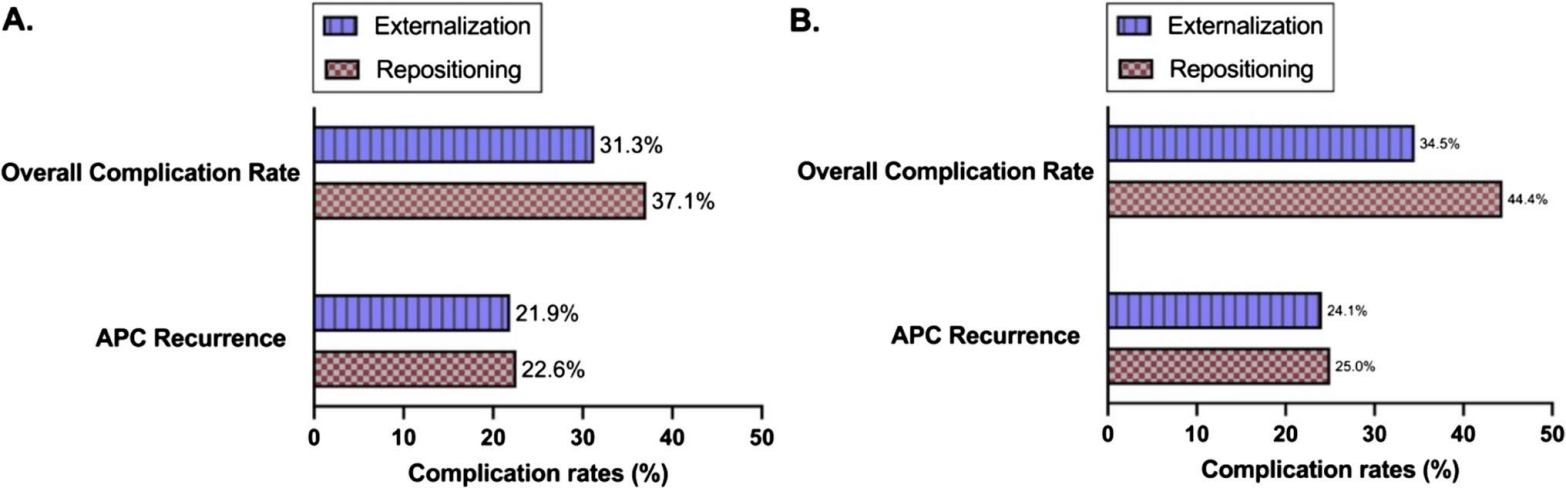
Complication rates for externalization and repositioning in the management of sterile abdominal pseudocysts. **A** The compiled data of studies at all time points. **B** Data from studies with a follow-up of 6 months or longer. Externalization is represented by blue-lined bars and repositioned by red checkered bars. On the *y*-axis, overall complication rates (which include APC recurrence, further shunt revisions, switching to a different shunt type, or shunt infection) and APC recurrence are depicted. On the *x*-axis are complication rates as a percentage. Fisher’s exact test determined no statistically significant difference in APC reoccurrence rates for the repositioning vs. externalization group (*p*-value = 0.99) and no statistical difference in overall complication rates (*p*-value = 0.57). A similar finding was demonstrated for data in studies with 6 or more months of follow-up with no statistically significant difference in APC reoccurrence rates for the repositioning vs. externalization group (*p*-value = 0.99) and no statistical difference in overall complication rates (*p*-value = 0.3861) (figure created with Prism GraphPad Software and BioRender)

Figure 3 plots the overall complication rates and APC reoccurrence rates. Fisher’s exact test determined that there was no statistically significant difference in APC reoccurrence rates for the repositioning vs. externalization group (*p*-value = 0.99). Fisher’s exact test also determined that there was no statistical difference in overall complication rates between the two groups (*p*-value = 0.57).

## Discussion

**Fig. 4:**
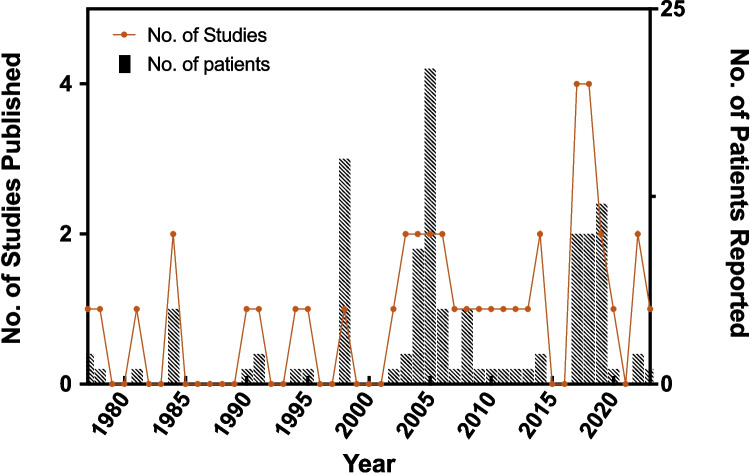
Trends in published articles on the management of sterile abdominal pseudocyst in the literature from 1977 to 2023. Year in intervals of 5 is represented on the *x*-axis, number of studies published is on the left *y*-axis, and number of patients reported in those studies is on the right *y*-axis. The orange line represents trends in the number of studies published, with each circle representing the number of studies in one year. Blue bars represent the number of patients included in those studies per year (figure created with Prism GraphPad Software)

APCs are one of the common abdominal complications related to VPS, which have been reported with an incidence rate from 0.25 to 10%. Traditional management options for these pseudocysts have been to either externalize with subsequent reinternalization or reposition the distal shunt in the peritoneum. The results of our analysis indicate there is no significant difference in complication rates between externalization and repositioning of the distal shunt catheter for the treatment of APCs. Of the articles we analyzed, few discussed or compared both techniques, and all of these were either case reports or case series [1, 14, 28, 32]. The limited number of studies analyzing these techniques in the context of sterile APCs highlights the utility of this review. This is further emphasized by the graph depicting trends in the number of studies for the management of sterile abdominal pseudocysts since 1977 (Fig. 4). There is a rising trend as it relates to published studies since the early 2000s. However, the number of patients each year has remained relatively steady with some year-to-year variation. Regardless of the rising trend in published articles regarding this topic, the studies are rare and continue to primarily consist of case reports and case series.

In this systematic review of 43 articles, we analyzed primary data from patients who underwent either repositioning or externalization as the treatment modality for their sterile abdominal pseudocyst between the date of inception and 2023 to determine if repositioning has similar complication rates as the more typical externalization approach. The clinical efficacy of these techniques was assessed by quantifying the rate of pseudocyst reoccurrence and overall complication rate, which encapsulates APC recurrence, further shunt revisions, switching to a different shunt type, or shunt infection. We determined a pseudocyst reoccurrence rate of 22.6% for repositioning and 21.9% for externalization. Furthermore, the overall complication rate was 37.1% for repositioning and 31.3% for externalization. Overall, these two techniques have similar complication rates. There is a higher non-significant rate of pseudocyst recurrence and overall complications for the repositioning group. Furthermore, when only looking at studies with a follow-up of 6 or more months, a similar finding was demonstrated with no statistical significance in pseudocyst recurrence and overall complications between the two techniques. While it is difficult to make any significant conclusions due to low sample sizes and inconsistent follow-up reporting, future prospective studies comparing larger patient cohorts could reveal this difference to be more substantial.

Our current findings agree with a study by Whittemore et al., who analyzed primary pseudocysts treated with either externalization or repositioning and determined that sterile abdominal pseudocysts without signs of infection can be managed with laparoscopic repositioning, resulting in a shorter hospitalization time and modest increase in recurrence compared to shunt externalization [53]. The results from that study were not incorporated here as culture-positive and culture-negative APCs were not well delineated. Despite this, we believe that due to the novelty of the study and the concurrence of their findings with this systematic review, it warranted reporting.

### Study limitations

A primary limitation of this study is the lack of prospective data, as we found no reports directly comparing repositioning versus externalization. Without this comparative data, we are unable to draw a definitive conclusion regarding the clinical efficacy of these two approaches. Another notable limitation of this review is that most of the studies are case reports and case series, which remain the lowest level of evidence. Furthermore, it is important to note that the results found here may have been susceptible to publication bias, considering that case reports on failed distal shunt revisions are likely published less frequently than successful revisions. Thus, the prevalence of outcomes and complications reported here may not be entirely reflective of the true population outcomes. However, the limited number of published studies on abdominal pseudocyst management highlights the increasing utility of this systematic review in providing a synthesis of the current limited literature. We found that many primary papers did not always contain valuable clinical data such as long-term follow-up, follow-up timelines, or other outcome measures that may be important in determining the efficacy of each approach. Knowledge of the long-term clinical course of these patients could alter our findings and indicate differences in outcomes unavailable with the current data. Further in-depth analyses that segregate patient populations by age group, prior complication rates, and longer follow-ups could provide insights and thus enhance the ability of neurosurgeons to conduct successful and safe management of sterile APCs. It should be cautioned that a comprehensive assessment of individual patient circumstances should be considered, regardless of the data presented here as for some patients with an extensive history of non-sterile APCs or shunt infections, externalization may be the preferred approach.

It is important to point out that more modern investigations of shunt material have improved the identification of pathogens. Sonication has been demonstrated to significantly enhance pathogen detection in VP-shunt infections, particularly in cases involving prior antibiotic treatment or low-virulent organisms [54]. It has also been shown to increase microbiological yield even in chronic or silent infections, where standard diagnostics often fail. Its clinical integration has improved germ detection from sterile-appearing cyst fluids, such as for pathogens like Cutibacterium acnes. However, the data presented within this manuscript did not necessarily undergo this protocol, which may explain the higher rate of sterile abdominal pseudocysts.

## Data Availability

No datasets were generated or analysed during the current study.
